# Effect of tumor and normal lung volumes on the lung volume–dose parameters of IMRT in non–small-cell lung cancer

**DOI:** 10.6061/clinics/2021/e2769

**Published:** 2021-06-29

**Authors:** Xi Zou, Linzhen Lan, Lijing Zheng, Jinmei Chen, Feibao Guo, Chuanshu Cai, Jinsheng Hong, Weijian Zhang

**Affiliations:** I Department of Radiotherapy, Cancer Center, First Affiliated Hospital of Fujian Medical University, Taijiang District, Fuzhou City, Fujian Province, 350005, China.; II Key Laboratory of Radiation Biology of Fujian Higher Education Institutions, the First Affiliated Hospital, Fujian Medical University, Taijiang District, Fuzhou City, Fujian Province, 350005, China.; III Department of Oncology, Fujian Provincial Hospital, Gulou District, Fuzhou City, Fujian Province, 350005, China.

**Keywords:** Non-Small-Cell Lung Cancer, Intensity-Modulated Radiotherapy, Volume-Dose Parameter, Lung Volume, Primary Tumor Volume

## Abstract

**OBJECTIVES:**

To explore the effect of tumor and normal lung volumes on lung volume-dose parameters in patients with non-small-cell lung cancer (NSCLC) who had undergone intensity-modulated radiation therapy (IMRT).

**METHODS:**

The clinical data of 208 patients with NSCLC who underwent radical IMRT between June 2014 and June 2018 were retrospectively analyzed. A regression model curve was used to evaluate the effect of tumor and normal lung volumes on normal lung relative volumes receiving greater than 5 and 20 Gy (V5, V20), on mean lung dose (MLD), and on absolute volumes spared from greater than 5 and 20 Gy (AVS5, AVS20).

**RESULTS:**

The V5, V20, and MLD of the bilateral lung were fitted to a quadratic equation curve with the change in tumor volume, which increased initially and then decreased when the tumor volume increased. The V5, V20, and MLD of the lung reached their apex when the tumor volumes were 288.07, 341.69, and 326.83 cm^3^, respectively. AVS5 and AVS20 decreased in a logarithmic curve with an increase in tumor volume. The V5, V20, and MLD of the small normal lung volume group were all significantly higher than those of the large normal lung volume group (*p*<0.001, *p*=0.004, *p*=0.002). However, the AVS5 and AVS20 of the small normal lung volume group were all significantly lower than those of the large normal lung volume group (*p*<0.001).

**CONCLUSION:**

The effects of tumor volume and normal lung volume on dose-volume parameters should be considered. AVS5 is an important supplementary dose limitation parameter for patients whose tumor volume exceeds a certain boundary value (approximately 300 cm^3^).

## INTRODUCTION

Radiation-induced lung injury (RILI) is the major factor contributing to dose-limiting toxicity following intensity-modulated radiotherapy (IMRT) in lung cancer. In clinical applications, the V5 and V20 (Vdose: fractional volume percent of the lung receiving a dose higher than XGy), as well as the mean lung dose (MLD), of the bilateral lung are controlled to reduce the risk of RILI (1). However, RILI still occurs at a high rate even when these parameters are strictly controlled (2). Recent studies have suggested that the sparing of the normal lung absolute volume using a particular level of low-dose irradiation (AVSx) in the bilateral lung should also be controlled during radiotherapy planning (3,4,5). Our recent study suggested that the AVS_5_ of the ipsilateral lung was prognostic for grade ≥2 RILI in patients with lung cancer following IMRT (6). Matsuo et al. (7) and Baker et al. (8) demonstrated that tumor volume was a predictor of radioactive pneumonia. Zhang et al. (9) described the influence of tumor volume in lung cancer on the volume parameters of normal lung tissue. However, the results of this study were based on models and single tumor morphologies and exhibited certain limitations. Therefore, the present study analyzed the applications of real IMRT in patients with lung cancer tumors. The main aim of this study was to analyze the influence of tumor volume and normal lung volume on lung dosimetry parameters and to provide a reference for dose-volume parameter evaluation in clinical applications.

## METHODS

### Patients and clinicopathological features

A retrospective analysis was performed on the medical records of 208 patients with lung cancer. The patients were treated between June 2014 and June 2018. The inclusion criteria were as follows: (i) patients with pathologically confirmed non-small-cell lung cancer (NSCLC), (ii) patients who received IMRT for radical radiotherapy because of local late operation inability, patients with early operation inability, or patients refusing surgical treatment for various reasons, (iii) intact case data and radiotherapy plan data with no serious lung disease, and (iv) N staging of zero to one. Patients were excluded if they had undergone previous (i) lung surgery or radiotherapy before additional radiotherapy, (ii) if they had undergone nonradical radiotherapy (radiotherapy dose <60 Gy), or (iii) if they had lung tumors with an N stage of two to three.

### Treatment planning

All patients underwent five-beam 6 MV X-ray IMRT. The radiation was delivered by a linear accelerator (Clinac 600C/D or Trilogy5918; Varian Medical Systems, Palo Alto, CA, USA), with one fraction per day and five fractions per week. The prescription dose was as follows: planning target volume (PTV) for gross target volume (GTV): 60-66 Gy/27-33 f, 2.00-2.25 Gy/f, clinical PTV: 48.0-54.0 Gy/27-33 f, 1.6-2.0 Gy/f. The dose limits for the organs at risk were as follows: bilateral lung, V20 ≤37% and MLD ≤20 Gy; spinal cord, maximum dose ≤45 Gy; heart, V40 ≤40%; and esophagus, V50 ≤50%. The lung was defined to exclude the gross GTV. The collapsed cone convolution was selected for the treatment planning.

### Dosimetric indicators

A dose-volume histogram related to the treatment planning system (Pinnacle3; Philips Medical Systems, Andover MA) was used to calculate the normal lung volume receiving >5, 10, 20, and 30 Gy (V5, V10, V20, and V30, respectively), MLD, and absolute volumes spared from >5, 10, 20, and 30 Gy (AVS5, AVS10, AVS20, and AVS30, respectively) of the bilateral lungs.

### Statistical analyses

Curve fitting: SPSS 21.0 software was used to fit the curve of GTV with total lung volumes V5 and V20 and the total MLD, AVS5, and AVS20. Data were analyzed using a regression model. The specific fitting method was selected according to the size of the R2 value, and 208 cases of patients with NSCLC were analyzed with regard to their lung volume (definition of lung volume: tissues were collected from 208 patients with NSCLC and were divided into large and small lung specimens with a normal lung volume mean of 3,145 cm^3^). The normal lung volume was subtracted from the total lung volume by GTV. The GTV of each group was fitted to the regression model according to bilateral lung V5, bilateral lung V20, bilateral lung MLD, bilateral lung AVS5, and bilateral lung AVS20.

Correlation analysis: The Wilcoxon test was used to analyze the effects of tumor morphology, tumor location, and lung volume on the lung dose-volume parameters (V5, V20, MLD, AVS5, AVS20). All data in this study were processed using Microsoft Office Excel 2007. SPSS 21.0 was used for statistical analyses. Statistical significance was set at *p*<0.05 (*p*<0.05).

## RESULTS

### Case characteristics

A total of 208 consecutive cases of NSCLC treated in our hospital between June 2009 and June 2016 were collected. Table 1 shows their clinicopathological characteristics.

### Curve fitting of GTV and dose-volume parameters

**Figure 1:**
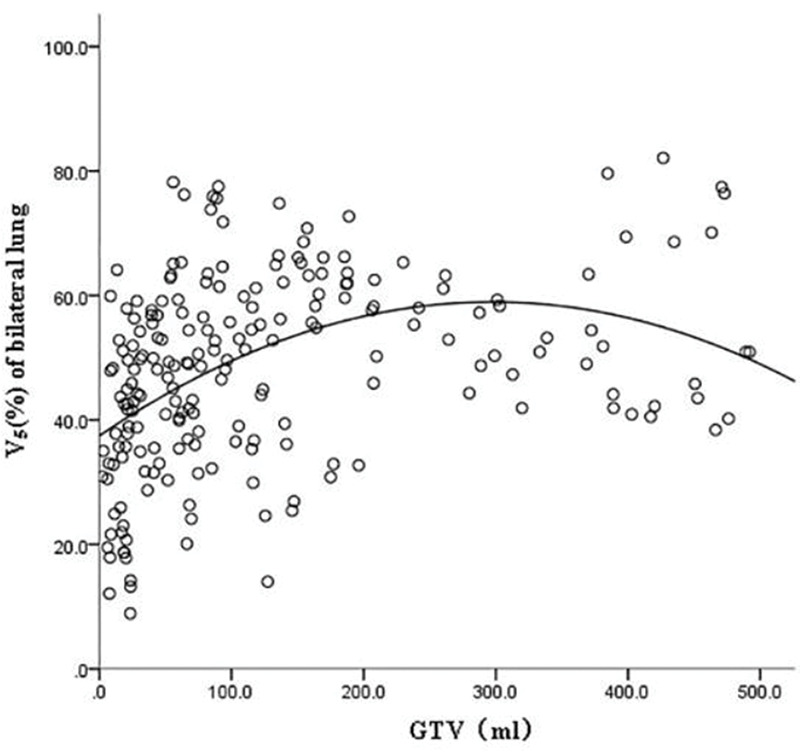
Fitting curve of GTV (mL) and bilateral lung V5 (%).

**Figure 2:**
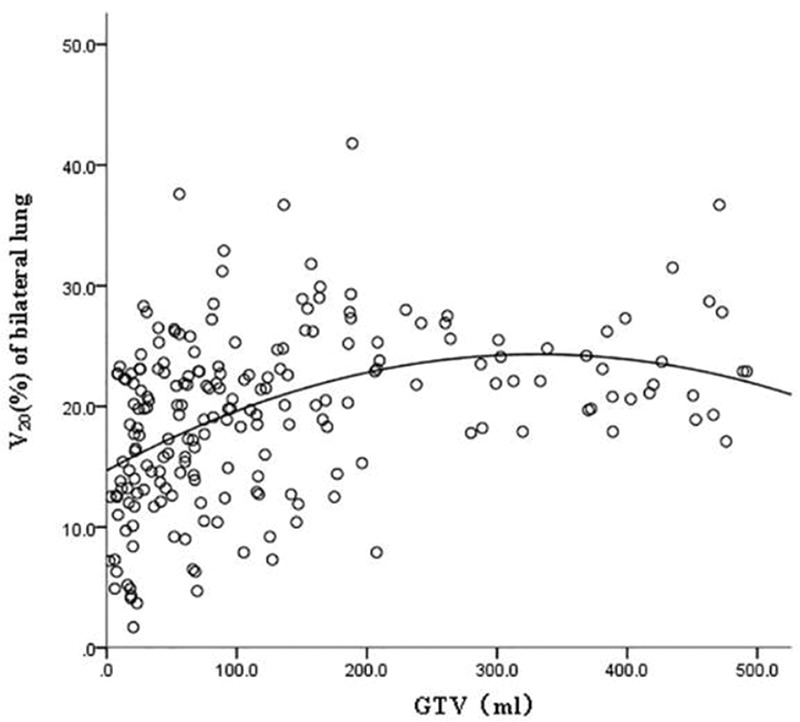
Fitting curve of GTV (mL) and bilateral lung V20 (%).

**Figure 3:**
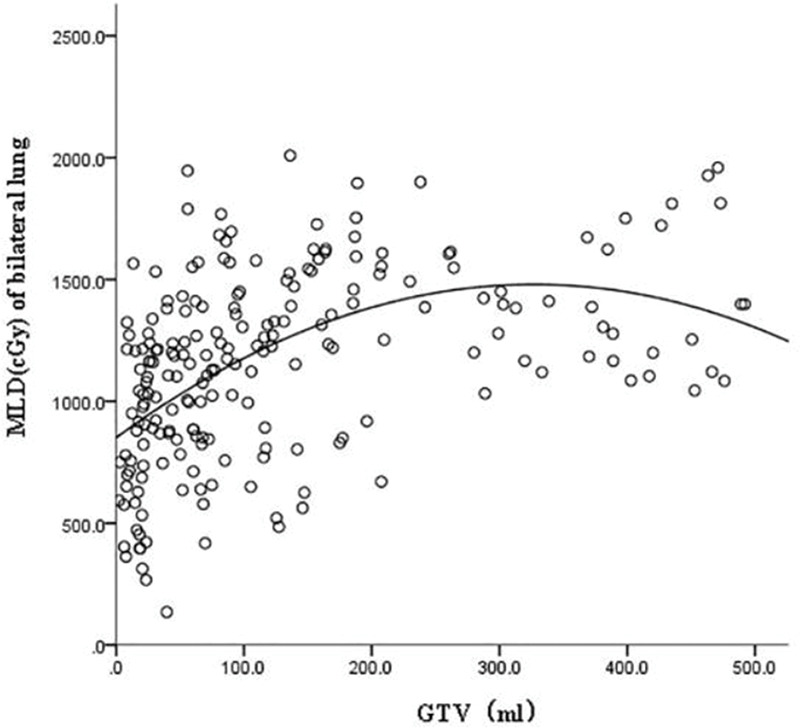
Fitting curve of GTV (mL) and bilateral lung MLD (cGy).

**Figure 4:**
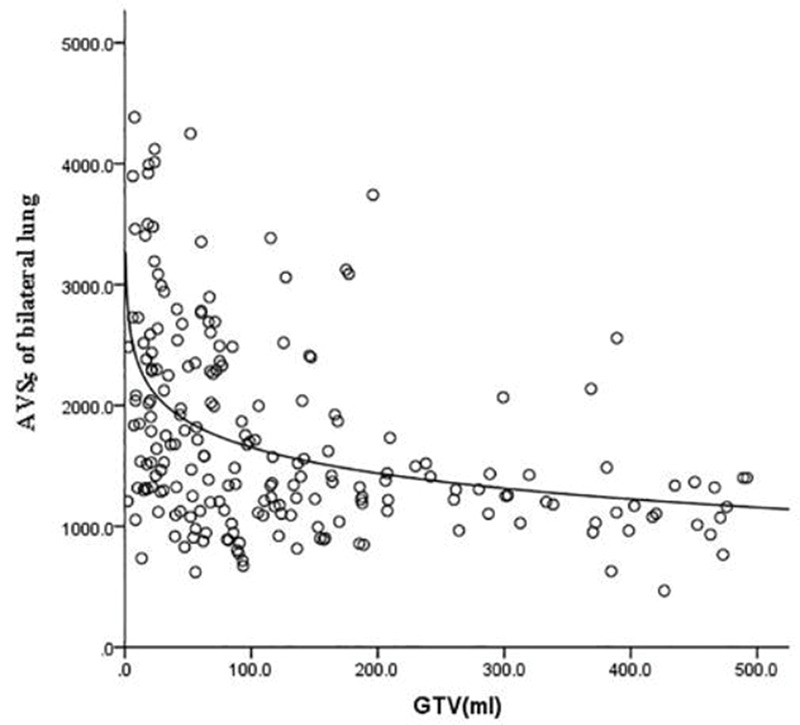
Fitting curve of GTV (mL) and bilateral lung AVS5.

**Figure 5:**
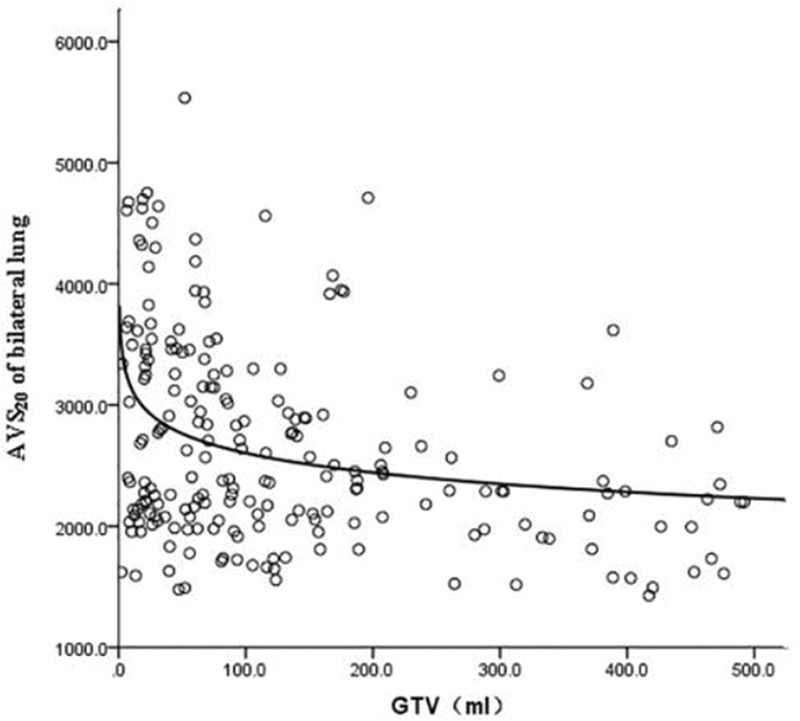
Fitting curve of GTV (mL) and bilateral lung AVS20.

GTV was fitted to the regression model with the following parameters: bilateral lung V5, bilateral lung V20, bilateral lung MLD, bilateral lung AVS5, and bilateral lung AVS20. The fitting curves of GTV with bilateral lung V5, bilateral lung V20, and bilateral lung MLD produced the highest R^2^ values. GTV with bilateral lung AVS5 and AVS20 exhibited a logarithmic curve, and Table 2 and Figures 1-5 show the results.

### Analysis of the regular pattern of the fitting curve

**Figure 6:**
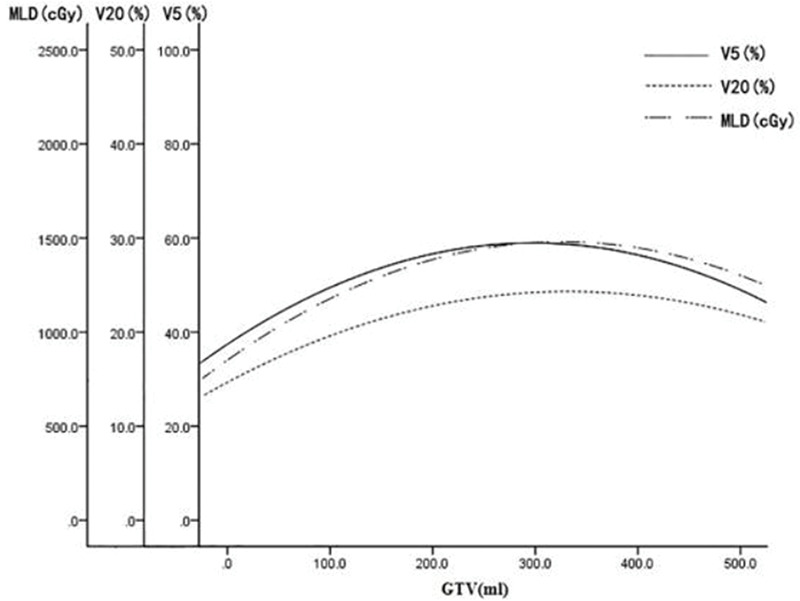
Fitting curve of GTV (mL) and bilateral lung V5, V20, and MLD.

The fitting curve of GTV with bilateral lung V5, bilateral lung V20, and bilateral lung MLD were performed using the quadratic equation. The three curves that were produced were similar; the equation of the fitting curves used the derivative, and the maximum value of the corresponding dose parameters was obtained. When GTV was in a certain range, bilateral lung V5, bilateral lung V20, and bilateral lung MLD increased with an increase in GTV. Bilateral lung V5, bilateral lung V20, and bilateral lung MLD reached the maximum value and did not increase further when GTVs were 288.07, 341.69, and 326.83 cm^3^, respectively (Figure 6).

**Figure 7:**
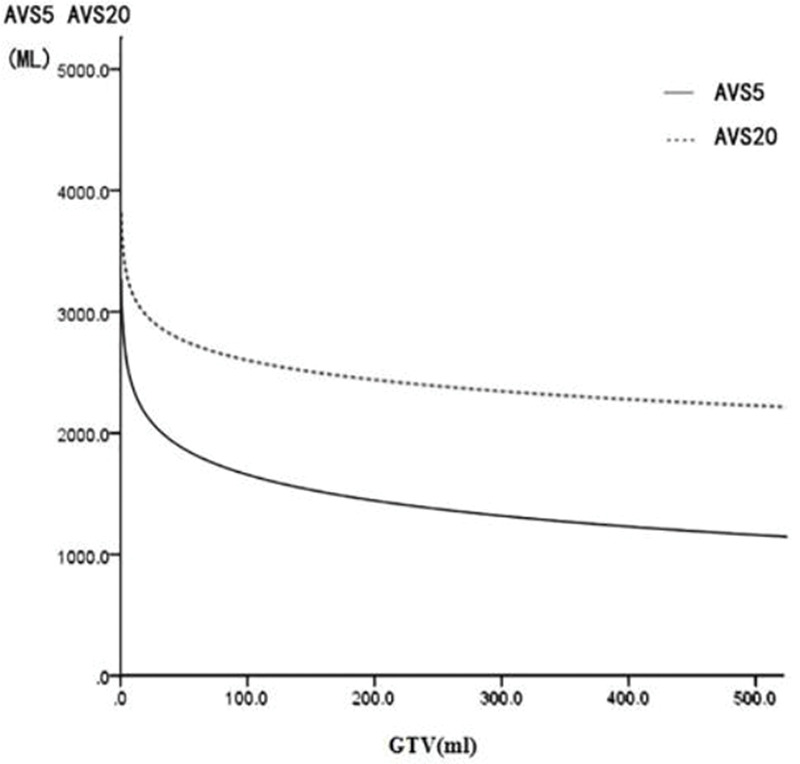
Fitting curve of GTV (mL) and bilateral lung AVS5 and AVS20.

The fitting of GTV with the bilateral lung AVS5 and bilateral lung AVS20 curves was in agreement with the regular pattern of the logarithmic regression equation. When GTV was within a certain range, bilateral lung AVS5 and bilateral lung AVS20 decreased with an increase in GTV. When GTV reached 300 cm^3^, bilateral lung AVS5 and bilateral lung AVS20 were 1,313.94 and 2,348.46 cm^3^ (bilateral lung AVS20/bilateral lung AVS5=1.8), respectively (Table 3). When GTV exceeded 300 cm^3^, bilateral lung AVS5 and bilateral lung AVS20 were stable (Figures 4-5 and 7).

### Difference between large and small normal lung volumes on the curve fitting of GTV and dose-volume parameters

**Figure 8:**
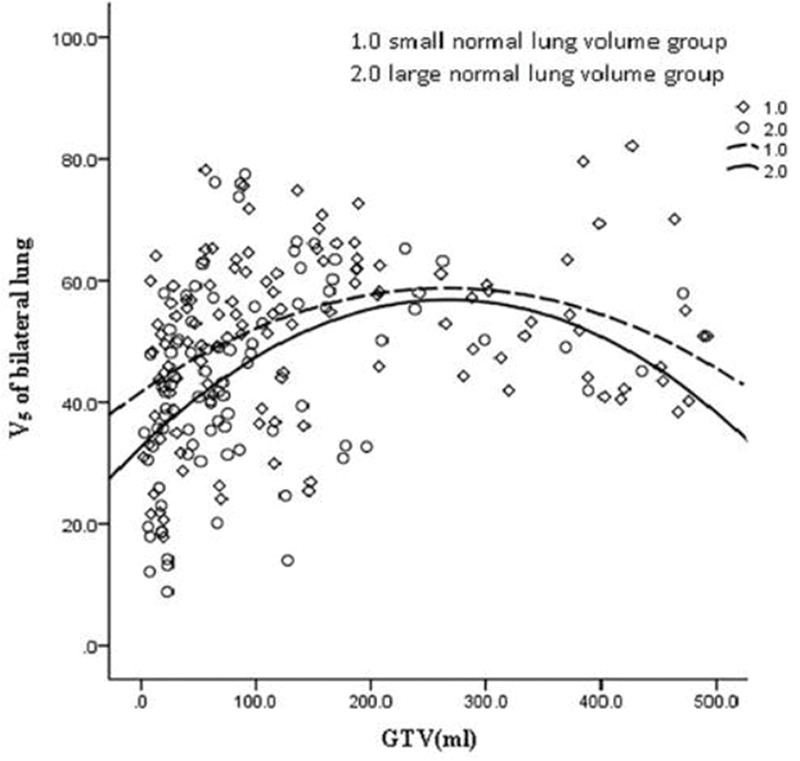
Fitting curve of GTV (mL) and bilateral lung V5 in the large and small volume groups.

**Figure 9:**
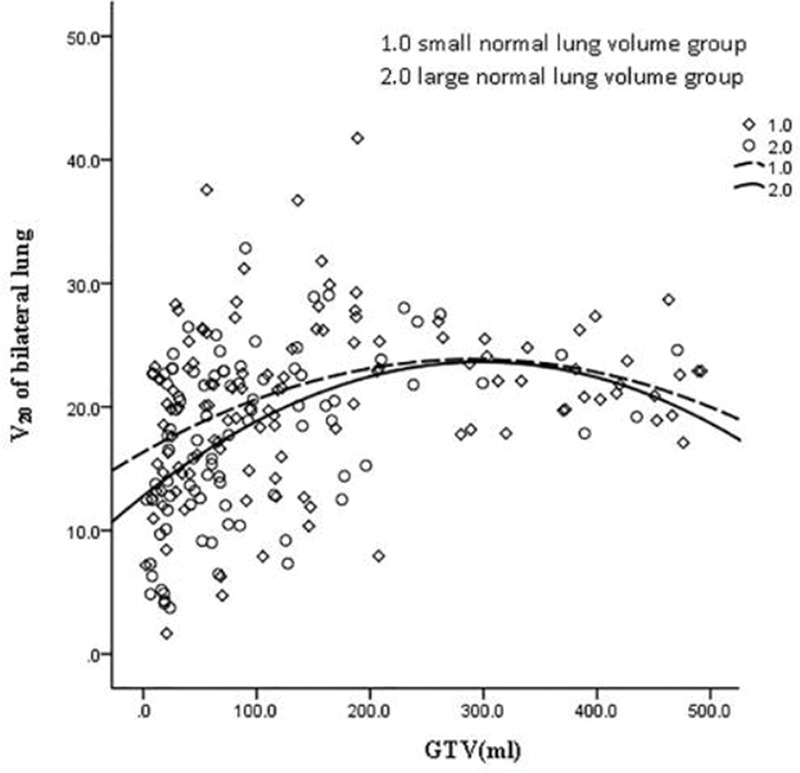
Fitting curve of GTV (mL) and bilateral lung V20 in the large and small volume groups.

**Figure 10:**
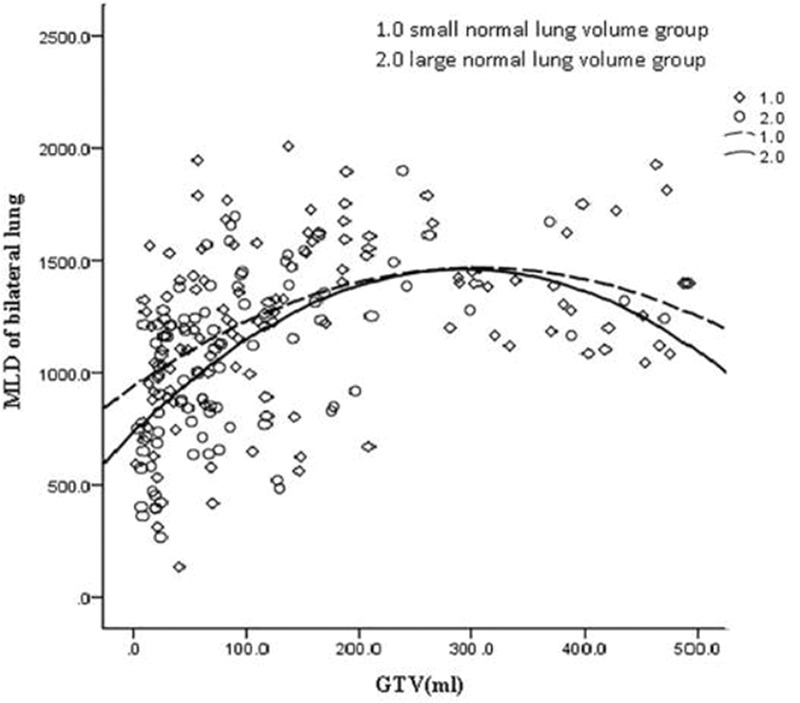
Fitting curve of GTV (mL) and bilateral lung MLD in the large and small volume groups.

**Figure 11:**
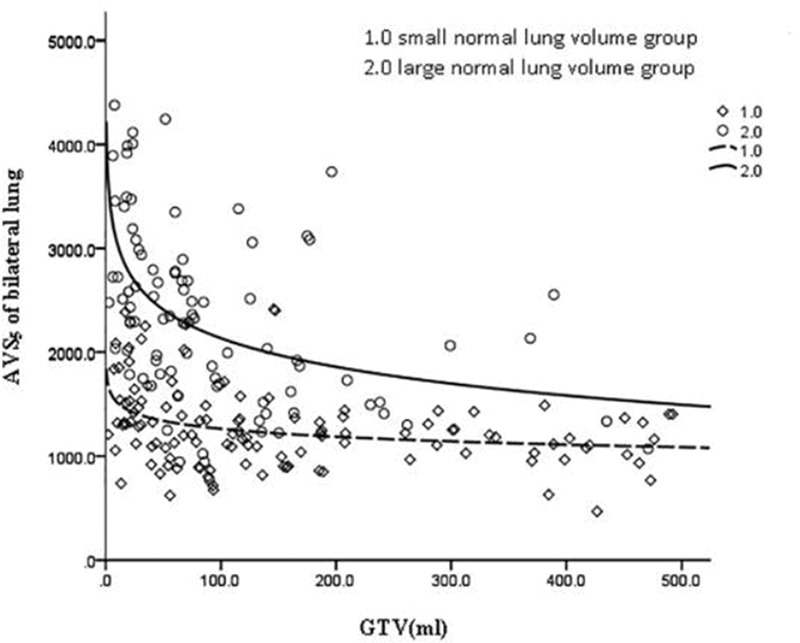
Fitting curve of GTV (mL) and bilateral lung AVS5 in the large and small volume groups.

**Figure 12:**
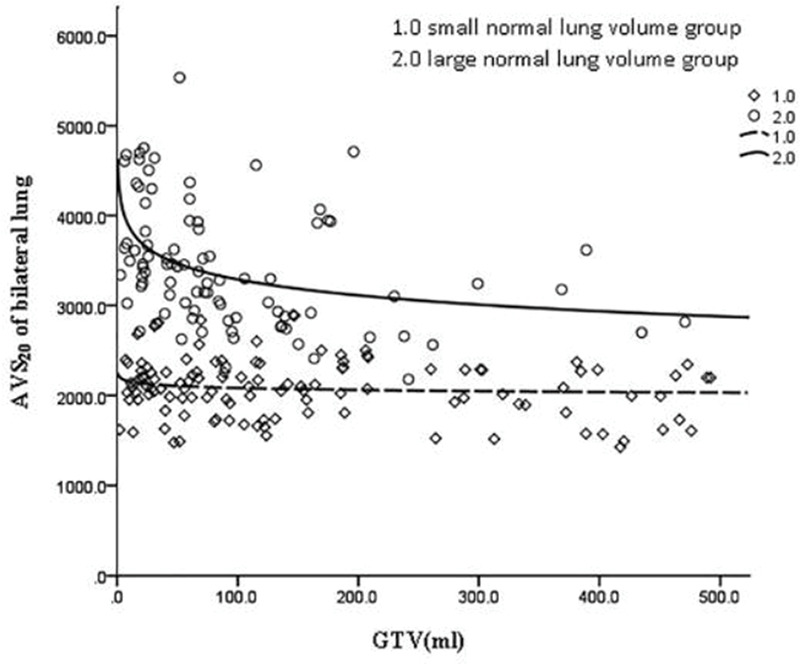
Fitting curve of GTV (mL) and bilateral lung AVS20 in the large and small.

The volume of the normal lung in this study was in the range of 1,746.6 to 6,093.6 cm^3^. The patients were divided into two groups with a mean volume of 3,145 cm^3^. The cases were divided into the large lung group (90 cases) and small lung group (118 cases). These two groups of GTV cases were fitted with dose-volume parameters (Figures 8-12). When the volume of GTV was equal between the two groups, parameters bilateral lung V5, bilateral lung V20, and bilateral lung MLD were higher in the small volume group than in the large volume group. The absolute volumes of the lung spared from receiving a dose higher than XGy (>XGy), such as AVS5 and AVS20, were significantly lower in the small group than in the large group.

### Pulmonary dose-volume parameters in the large and small volume groups

The lung volume parameters of the large and small volume groups were compared. The mean squared deviation indicated a large difference between the samples and data fitted in the nonnormal distribution (Table 4).

### Nonparametric analysis of the lung volume and its influence on lung dose-volume parameters

The average number of lung dose-volume parameters of the two groups of cases indicated that the difference in sample size was large and that the data followed a nonnormal distribution. The difference between the parameters of the lung volume and volume parameters of the lung were analyzed using the Wilcoxon test (Table 5). The results indicated that the effects of lung volume on dose-volume parameters V5, V20, MLD, AVS5, and AVS20 were statistically significant, notably with regard to V5, AVS5, and AVS20. When the tumor volume was equal between the two groups, a protective effect was observed in normal lung tissue.

## DISCUSSION

Moderate and severe radiation pneumonia not only leads to the interruption of radiation therapy but also affects the therapeutic effect and quality of life of the patients. This disease can further lead to mortality; therefore, considerable efforts have been made to prevent it. This study demonstrated that the incidence of a grade higher than and/or equal to RILI (≥2 RILI) was significantly lower when the AVS5 of the ipsilateral lung was higher than and/or equal to 564.9 cm^3^ (≥564.9 cm^3^) than when the AVS_5_ was lower than 564.9 cm^3^(<564.9 cm^3^) (*p*=0.008). The low-dose irradiation relative volumes and MLD of the bilateral or ipsilateral lung were associated with a grade≥2 RILI (grade≥2 RILI), whereas the AVS5 of the ipsilateral lung was prognostic for grade≥2 RILI for lung cancer following IMRT (6). Briere et al. demonstrated that in 579 NSCLC cases of three-dimensional conformal radiotherapy or IMRT, 38% of the patients had an AVS_40_ value lower than 1,854 cm^3^ (AVS40 <1,854 cm^3^). A total of 72 patients in this cohort exhibited radiation pneumonitis 6 months after radiotherapy. The probability of radiation pneumonitis at 6 months following radiotherapy (507 cases) was 12%, which was lower than that in the low AVS40 group (*p*<0.001) (3). Tsujino et al. (4) conducted a retrospective analysis of 122 patients with NSCLC receiving concurrent chemoradiotherapy and demonstrated that an AVS5 <1,500 cm^3^ (AVS5 <1,500 cm^3^) was an independent risk factor for three or more levels of radionuclide pneumonia. Therefore, in addition to examining pulmonary V5, V20, and MLD, AVS5 restrictions should be considered in the establishment of IMRT for NSCLC (10).

Our previous studies have found that tumor shape and location have obvious effects on dose-volume parameters (11). Briere et al. (3) demonstrated that lung volume is an important predictor of patients with radiation pneumonitis. Among these patients, 34% with an AVS_40_ <2,533 cm^3^ (n=79) exhibited radiation pneumonitis at 6 months after radiotherapy. The average normal lung volume was 3,701 cm^3^, whereas the patients with an AVS_40_ >2,533 cm^3^ (500 cases) had only 13% radiation pneumonitis. The average normal lung volume was 2,232 cm^3^. The difference was statistically significant (*p*<0.001). In the present study, 208 cases of NSCLC with a moderate volume of 3,145 cm^3^ were divided into the small volume group (118 cases) and mass group (90 cases) to analyze the volume parameters of the lung. The results indicated that when the tumor volume was equal, the volume ratio of the normal lung tissue in the large volume group (>3,145 cm^3^) was higher than small volume group. The average dose of the bilateral lung was lower than that of the small volume group (<3,145 cm^3^) (*p*<0.001, *p*=0.004, *p*=0.002). The absolute volume of the spared lung that received a dose higher than XGy (>XGy), such as in the cases of AVS_5_ and AVS_20,_ was significantly higher than that of the small volume group (*p*<0.001). As a “parallel tissue,” the lung should theoretically have a normal boundary value of the normal lung volume to compensate for the respiratory function. A larger normal lung volume within a certain range is correlated with the greater absolute volume of the lung that can be spared from irradiation and smaller possibility for development of radiation pneumonitis. Therefore, in patients with a smaller normal lung size, AVS_x_ must be examined before the formulation of a treatment plan.

This study has the following limitations: relatively small sample size, low number of cases with tumor volume >300 mL, grouping of N1 cases, and lack of grouping in the specimens collected from the left and right parts of the lung. These disadvantages affected the curve fitting and statistical results to a certain extent. The sample size could be further expanded to verify the overall conclusions. In this study, the effect of tumor size or normal pulmonary volume on the dose-volume parameters of the lung was based on a pure data study model. Additional studies can further examine the actual occurrence of patients with radiation pneumonia and verify the validity of the overall conclusion.

## CONCLUSION

The correlation between the tumor volume and sparing of the normal lung absolute volume was notably noted at low-dose irradiation (AVSx). During lung cancer radiotherapy, the use of AVS5 and AVS20 and combination of Vx and MLD to predict the occurrence of RILI may be more reliable in cases of large tumor volumes. The parameters of bilateral lung V5, V20, and MLD in the small volume group were higher than those in the large volume group, whereas AVS5 and AVS20 were lower in the large volume group than in the small volume group. It is evident that the probability of RILI in the small volume group is higher than that in the large volume group. In the formulation of the treatment plan, the normal lung tissue of a certain volume should be protected. Therefore, the absolute volume of the lungs should be carefully considered.

## AUTHOR CONTRIBUTIONS

All authors read and approved the final version of the manuscript for publication. Zou X and Lan L contributed equally to the data curation, literature review, and manuscript drafting, review and editing. Zheng L contributed significantly to data acquisition, data analysis, and manuscript preparation. Chen J and Guo F acquired data and played an important role in interpreting the results. Cai C helped performing the analysis with constructive discussions. Hong J and Zhang W conceived and designed the experiments, revised the manuscript, and approved the final version of the manuscript.

## Figures and Tables

**Table 1:** General clinicopathological characteristics of 208 cases of NSCLC.

Factor	Case (%)	Factor	Case (%)
Sex		Age (year)	
Male	174 (83.7)	≤60	114 (54.8)
Female	34 (16.3)	>60	94 (45.2)
Pre-RT treatment		Primary tumor volume	
chemotherapy	135 (64.9)	≥100 cm^3^	89 (42.8)
TKI	39 (18.8)	<100 cm^3^	119 (57.2)
none	34 (16.3)	Tumor site	
Location		Upper lung	103 (49.5)
Central	147 (70.7)	Lower lung	105 (50.5)
Peripheral	61 (29.3)	Tumor morphology	
Other	8 (3.8)	Transverse strip	81 (38.9)
Pathological type		Longitudinal bar	127 (61.1)
Squamous	105 (50.5)	Pulmonary volume	
Adenocarcinoma	95 (45.7)	Small volume of lung	118 (56.7)
Other	8 (3.8)	Large volume lung	90 (43.3)

TKI = epidermal growth factor receptor-tyrosine kinase inhibitor.

**Table 2:** Curve fitting results of GTV and lung dose-volume parameters in 208 cases of NSCLC.

Dose-volume parameter of the lung	Decisive coefficient (r^2^)	*p*	Tumor volume corresponding to the highest point of the curve
V5 of bilateral lung	0.180	<0.001	288.07
V20 of bilateral lung	0.181	<0.001	341.69
MLD of bilateral lung	0.255	<0.001	326.83
AVS5 of bilateral lung	0.181	<0.001	/
AVS20 of bilateral lung	0.100	<0.001	/

Note: By using the curve fitting method, the tumor volume and whole lung V5, V20, and MLD were fitted with two equations (the specific fitting method was selected according to the size of the R^2^ value), and the maximum value was obtained according to the equation of the fitting curve. The tumor volume and whole lung AVS5 and AVS20 were fitted using the logarithmic equation.

**Table 3:** The fitting curve equation when GTV = 300 mL corresponds to the lung dose-volume parameter.

Volume dose parameters	Corresponding value when GTV = 300 mL	Volume dose parameters	Corresponding value when GTV = 300 mL
V5 of bilateral lung	57.57%	AVS5 of bilateral lung	1313.94 cm^3^
V20 of bilateral lung	24.78%	AVS20 of bilateral lung	2348.46 cm^3^
MLD of bilateral lung	1476 cGy		

**Table 4:** Parameters of the 208 cases comprising the large and small volume NSCLC groups.

	Small volume group			Large volume group		
	Case	x±s	Median	Case	x±s	Median
bilateral lung V5 (%)	118	51.00±13.72	52.75	90	43.67±15.59	43.03
bilateral lung V20 (%)	118	20.47±6.60	19.72	90	17.47±6.76	18.68
bilateral lung MLD (cGy)	118	1222.75±365.15	1222.85	90	1052.21±371.74	1124.90
bilateral lung AVS5 (cm^3^)	118	1269.63±385.46	1214.48	90	2352.88±842.93	2311.17
bilateral lung AVS20 (cm^3^)	118	2086.65±322.20	2371.60	90	3428.57±673.01	3308.92

**Table 5:** Comparison of the influence of lung volume on lung dose-volume parameters by Wilcoxon test.

	Small volume group M (P25, P75)	Large volume group M (P25, P75)	Z	*p*
bilateral lung V5 (%)	52.75 (42.14, 61.12)	43.03 (32.97, 55.63)	-3.058	<0.001
bilateral lung V20 (%)	19.72 (16.53, 24.73)	18.68 (12.50, 22.73)	-2.850	0.004
bilateral lung MLD (cGy)	1222.85 (1012.43, 1474.88)	1124.90 (766.8, 1315.60)	-3.092	0.002
bilateral lung AVS5 (cm^3^)	1214.48 (1023.10, 1427.24)	2311.17 (1724.15, 2821.16)	-9.545	<0.001
bilateral lung AVS20 (cm^3^)	2371.60 (1904.28, 2287.83)	3308.92 (2902.97, 3865.97)	-11.94	<0.001

## References

[1] National Comprehensive Cancer Network (2020). Non-Small Cell Lung Cancer Version v.1.

[2] Jiang ZQ, Yang K, Komaki R, Wei X, Tucker SL, Zhuang Y (2012). Long-term clinical outcome of intensity-modulated radiotherapy for inoperable non-small cell lung cancer: the MD Anderson experience. Int J Radiat Oncol Biol Phys.

[3] Briere TM, Krafft S, Liao Z, Martel MK (2016). Lung Size and the Risk of Radiation Pneumonitis. Int J Radiat Oncol Biol Phys.

[4] Tsujino K, Hashimoto T, Shimada T, Yoden E, Fujii O, Ota Y (2014). Combined analysis of V20, VS5, pulmonary fibrosis score on baseline computed tomography, and patient age improves prediction of severe radiation pneumonitis after concurrent chemoradiotherapy for locally advanced non-small-cell lung cancer. J Thorac Oncol.

[5] Jenkins P, Watts J (2011). An improved model for predicting radiation pneumonitis incorporating clinical and dosimetric variables. Int J Radiat Oncol Biol Phys.

[6] Chen J, Hong J, Zou X, Lv W, Guo F, Hong H (2015). Association between absolute volumes of lung spared from low-dose irradiation and radiation-induced lung injury after intensity-modulated radiotherapy in lung cancer: a retrospective analysis. J Radiat Res.

[7] Matsuo Y, Shibuya K, Nakamura M, Narabayashi M, Sakanaka K, Ueki N (2012). Dose--volume metrics associated with radiation pneumonitis after stereotactic body radiation therapy for lung cancer. Int J Radiat Oncol Biol Phys.

[8] Baker R, Han G, Sarangkasiri S, DeMarco M, Turke C, Stevens CW (2013). Clinical and dosimetric predictors of radiation pneumonitis in a large series of patients treated with stereotactic body radiation therapy to the lung. Int J Radiat Oncol Biol Phys.

[9] Zhang F, Zheng M, Chen J, Gao J (2009). Study of effect of lung tumor location and volume on dosimetric parameters using Alderson Rando phantom. Chinese Journal of Radiation Oncology.

[10] Zou X, Chen J, Hong J, Guo F, Lan L, Zhang W (2017). [Effect of tumor volume on pulmonary dose-volume parameter by intensity-modulated radiation therapy in non-small cell lung cancer]. Zhong Nan Da Xue Xue Bao Yi Xue Ban.

[11] Zou X, Zheng L, Chen J, Zhang W, Hong J, Guo F (2018). [Effect of tumor shape and location on lung volume-dose parameters of intensity-modulated radiation therapy for non-small cell lung cancer]. Chinese Journal of Radiation Oncology.

